# Functional role of an unusual tyrosine residue in the electron transfer chain of a prokaryotic (6–4) photolyase†
†Electronic supplementary information (ESI) available. See DOI: 10.1039/c7sc03386a


**DOI:** 10.1039/c7sc03386a

**Published:** 2017-12-11

**Authors:** Daniel Holub, Hongju Ma, Norbert Krauß, Tilman Lamparter, Marcus Elstner, Natacha Gillet

**Affiliations:** a Department for Theoretical Chemical Biology , Institute for Physical Chemistry , Karlsruhe Institute for Technology , Kaiserstr. 12 , 76131 , Karlsruhe , Germany . Email: natacha.gillet@kit.edu; b Botanical Institute , Karlsruhe Institute for Technology , Fritz Haber Weg 4 , 76131 , Karlsruhe , Germany; c Institute of Biological Interfaces (IGB2) , Karlsruhe Institute for Technology , Kaiserstr. 12 , 76131 , Karlsruhe , Germany

## Abstract

Cryptochromes and photolyases form a flavoprotein family in which the FAD chromophore undergoes light induced changes of its redox state. During this process, termed photoreduction, electrons flow from the surface *via* conserved amino acid residues to FAD. The bacterial (6–4) photolyase PhrB belongs to a phylogenetically ancient group. Photoreduction of PhrB differs from the typical pattern because the amino acid of the electron cascade next to FAD is a tyrosine (Tyr391), whereas photolyases and cryptochromes of other groups have a tryptophan as direct electron donor of FAD. Mutagenesis studies have identified Trp342 and Trp390 as essential for charge transfer. Trp342 is located at the periphery of PhrB while Trp390 connects Trp342 and Tyr391. The role of Tyr391, which lies between Trp390 and FAD, is however unclear as its replacement by phenylalanine did not block photoreduction. Experiments reported here, which replace Tyr391 by Ala, show that photoreduction is blocked, underlining the relevance of Tyr/Phe at position 391 and indicating that charge transfer occurs *via* the triad 391-390-342. This raises the question, why PhrB positions a tyrosine at this location, having a less favourable ionisation potential than tryptophan, which occurs at this position in many proteins of the photolyase/cryptochrome family. Tunnelling matrix calculations show that tyrosine or phenylalanine can be involved in a productive bridged electron transfer between FAD and Trp390, in line with experimental findings. Since replacement of Tyr391 by Trp resulted in loss of FAD and DMRL chromophores, electron transfer cannot be studied experimentally in this mutant, but calculations on a mutant model suggest that Trp might participate in the electron transfer cascade. Charge transfer simulations reveal an unusual stabilization of the positive charge on site 391 compared to other photolyases or cryptochromes. Water molecules near Tyr391 offer a polar environment which stabilizes the positive charge on this site, thereby lowering the energetic barrier intrinsic to tyrosine. This opens a second charge transfer channel in addition to tunnelling through the tyrosine barrier, based on hopping and therefore transient oxidation of Tyr391, which enables a fast charge transfer similar to proteins utilizing a tryptophan-triad. Our results suggest that evolution of the first site of the redox chain has just been possible by tuning the protein structure and environment to manage a downhill hole transfer process from FAD to solvent.

## Abstract 2

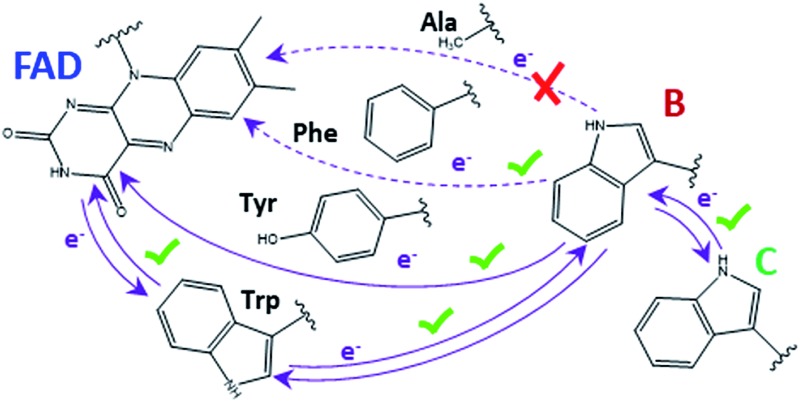
FAD photoreduction mechanism by different aromatic residues in a phylogenetically ancient photolyase.

## Introduction

Cryptochromes and photolyases are homologous proteins with a central flavin adenine dinucleotide (FAD) chromophore that fulfil different biological functions, which are most often triggered by light. During a process termed photoreduction, oxidised FAD of cryptochromes or photolyases takes up one or two electrons to convert to the semiquinone or the fully reduced state, respectively. In cryptochromes, which often function as photoreceptor proteins, FAD adopts the oxidised form in darkness. In these proteins the photoreduction is regarded as the first step of a signal transduction cascade.^1^ Cryptochrome of migrating birds functions as molecular compass, due to the radical pair formed in the semiquinone state. In photolyases, which are light triggered enzymes that repair UV-damaged DNA, the chromophore assumes the fully reduced form, FADH^–^, *in vivo* but converts to the semireduced or oxidised form under aerobic conditions *in vitro*. Photoreduction is important to ensure a high level of reduced FADH^–^, which is required for DNA repair.^2^–^4^

Because FAD is embedded in the centre of the protein, a direct transfer of electrons from solution is not possible. Crystal structures and site directed mutagenesis identified amino acids that constitute an electron transfer (ET) cascade. Most often, replacement of relevant Trp or Tyr by Phe results in a slower or blocked photoreduction. The interpretation of mutant results can however be hampered by the possibility of parallel pathways.^5^–^7^

**Fig. 1:**
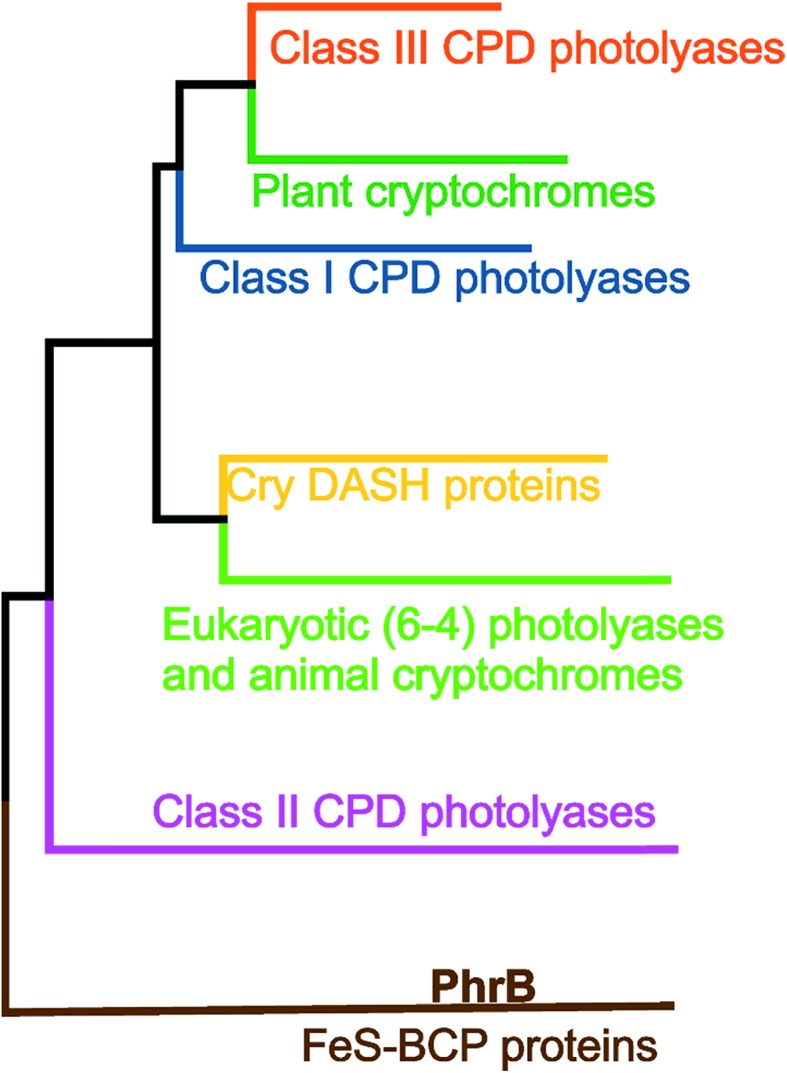
Phylogenetic tree with different classes of the cryptochrome–photolyase family.^11^

Three Trp residues at position 306, 382 and 359 of *E. coli* photolyase constitute the first identified ET cascade of the cryptochrome–photolyase family (PDB ; 1DNP).^8^ These Trp residues are conserved in all other members of class I CPD photolyases, to which *E. coli* photolyase belongs, in class III CPD photolyases, CRY-DASH proteins, plant cryptochromes, animal cryptochromes and eukaryotic 6–4 photolyases, but not in class II CPD photolyases^9^ or FeS-BCP proteins (see Fig. 1 for a phylogenetic tree of photolyases and cryptochromes). In class I and class III CPD photolyases, additional Trp residues have been shown to be involved in photoreduction. For example, in the class III CPD photolyase PhrA, the Trp residue of the classical triad that is closest to FAD is linked to a second, less conserved electron pathway comprising two Trp residues.^5^ The class II CPD photolyases have another Trp triad, which is conserved among this group.^10^

Trp side chains are chemically ideally suited for ET processes due to their aromatic cycles, low redox potential (around 0.6 V at pH 7)^12^ and stable radical state for the deprotonated form. However, in several CPD class I and II photolyases, Tyr residues are also involved in ET. In the class I CPD photolyase from *Anacystis nidulans*, a Tyr radical is formed within 50 μs after FAD excitation, as detected by ultrafast spectroscopy.^13^ In *Methanosarcina mazei* CPD class II photolyase, a Tyr residue is required for full photoreduction. In the *Xenopus laevis* (6–4) photolyase the involvement of a Tyr residue in photoreduction was shown by electron paramagnetic resonance.^14^

The conservation of Tyr or Trp for electron transfer shows that this selection is not a random process. Factors such as the chemical environment (other nearby amino acids or water) certainly play a significant role. Electron transferring Tyr residues are usually located close to the protein surface, surrounded by water and/or located close to deprotonating amino acids.^15^,^16^ Electron transferring Trp residues can occur at the periphery or in the centre of a protein.

**Fig. 2:**
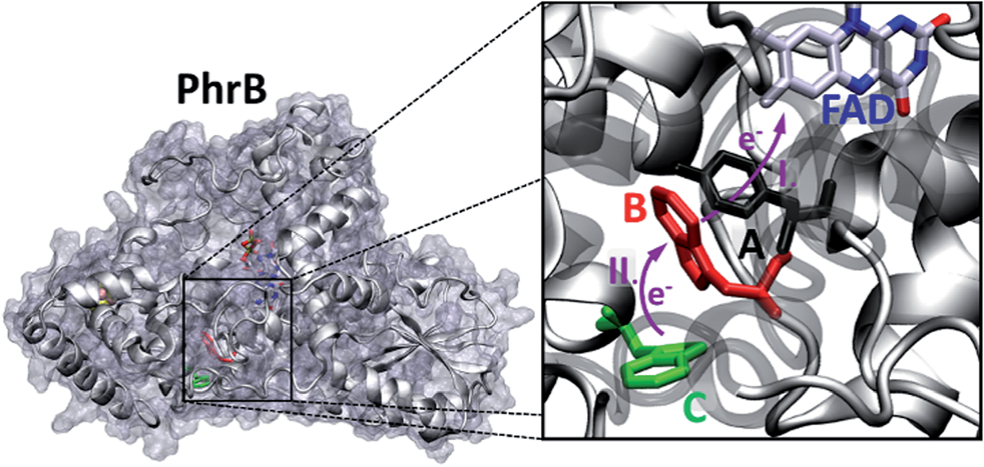
PhrB structure (PDB ID 4DJA) and charge migration pathway from the protein surface Trp342 (**C**, green) to FAD *via* Trp390 (**B**, red) and Tyr391 (**A**, black).^17^ Light induced consecutive electron transfers **B** → FAD* and **C** → **B** are indicated by purple arrows and labelled I and II, respectively. Images were rendered with VMD.^20^

The group of FeS-BCP proteins is a phylogenetically distinct group of (6–4) photolyases (Fig. 1) with unique properties such as an Fe–S cluster and a 6,7-dimethyl-8-ribityllumazine (DMRL) antenna chromophore.^17^ The ET in FeS-BCP proteins differs from all other cryptochrome and photolyase groups in several ways. In the FeS-PCB members PhrB from *Agrobacterium fabrum* (PDB ; 4DJA)^17^ and CryB from *Rhodobacter sphaeroides* (PDB ; 3ZXS)^18^ photoreduction proceeds *via* Trp390 and Trp342 (PhrB numbering), as shown by site directed mutagenesis. These residues are highly conserved in FeS-BCP members (see ESI Fig. S1†). In both proteins, the Tyr391 side chain is directly located between Trp390 and FAD (see also Fig. 2) suggesting that this Tyr must be part of the ET chain. However, when the Tyr was replaced by Phe, the photoreduction rate of PhrB was not affected,^19^ and only slightly affected in CryB.^18^ In about 30% of FeS-BCP proteins, a Phe is placed at this position (see Fig. S1†).

These observations raise several questions about Tyr391:

– is this residue involved in photoreduction as the first electron donor of FAD, as proposed by its spatial position?

– if yes, how can the electrons be transmitted *via* this Tyr residue and why is it a Tyr residue, whereas in other groups of photolyases and cryptochromes a Trp residue serves as electron donor for FAD?

– Phe is usually used to interrupt electron chains. Why does the replacement of Tyr391 by Phe not interrupt the electron chain?

FAD photoreduction involving a Trp triad has been widely studied by computational approaches in *Escherichia coli* photolyase,^21^–^23^ *Arabidopsis thaliana* cryptochrome,^24^–^29^ *Synechocystis* sp. CRY-DASH protein^30^ or *Xenopus laevis* (6–4) photolyase.^31^ These studies highlighted the role of Trp in the classical triad^27^–^29^ or of additional residues^30^,^31^ at an atomistic level, as well as the crucial role of the environment^24^,^25^,^29^ or the quantum effects in these ultrafast charge transfers.^26^ In our group, we have established a quantum mechanics/classical mechanics (QM/MM) scheme based on fragment orbital tight-binding density functional theory (FODFTB) coupled to MM molecular dynamics (MD).^22^–^24^,^32^ Our approach allows direct simulations of the charge propagation along a Trp triad. Previously we established our method by successful reproductions of ET rate constants in *E. coli* photolyase^23^ and *Arabidopsis* cryptochrome^24^ and also highlighted the role of environment in the downhill charge transfer process. Applying these techniques, we are able to shed light onto the molecular evolution of ET pathways in different proteins belonging to the cryptochrome–photolyase family.

In the present work, we investigate the role of Tyr391 in PhrB by experimental and QM/MM approaches, comparing PhrB wild type (WT) with its Y391F, Y391W and Y391A mutants. We regard that the analysis of the Tyr to Trp replacement is important for an understanding of the evolution of photolyases and cryptochromes, which most often have a Trp triad as electron transmission. Due to the difference in ionization potentials, it is expected that a Tyr residue slows down or blocks charge transfer, and that the replacement of Tyr by Trp would result in increased ET rates. However, all forward and backward charge transfers along the triad and subsequent possible charge recombination with FAD have to be considered. The comparison between PhrB and other members of the cryptochrome–photolyase family shed light on protein fine-tuning, enhancement of FAD photoreduction rates and avoidance of charge recombination.

## Experimental section

### Experimental methods

#### Site directed mutagenesis, protein expression and purification

A PhrB *E. coli* expression vector based on pET21b was used for recombinant expression of PhrB in ER2566 cells. The vectors for WT and the Y391F mutant are described in earlier publications.^19^,^33^ To obtain the Y391A and Y391W mutants, site directed mutagenesis was performed according to the Quik Change mutagenesis kit (Agilent) using a pair of complementary primers (Table S1†) with the desired mutation in the middle for initial polymerase reactions. Mutagenesis success and correctness of the sequences were confirmed by DNA sequencing. Expression and purification followed the procedure described in ref. 11 for WT and mutants. In brief, *E. coli* cells from agar plates were used for the inoculation of 3 l LB containing ampicillin. Following specific induction of recombinant expression with IPTG and subsequent incubation over night at 28 °C, all purification steps were carried out at 4 °C. Cells were harvested by centrifugation, suspended in 50 ml extraction buffer (50 mM Tris/HCl, 5 mM EDTA, 300 mM NaCl, 10% glycerol, pH 7.8) and extracted with a French Press (America Instrument Company) at 1000 bar. Following centrifugation and precipitation of soluble protein by ammonium sulfate (93% saturation), the protein pellet was suspended in EDTA free buffer. Soluble protein was purified by Ni affinity chromatography followed by size exclusion chromatography. The final buffer was 50 mM Tris/HCl, 5 mM EDTA, 300 mM NaCl, 10% glycerol, pH 7.8.

#### Photoreduction measurements by UV/vis spectroscopy

PhrB WT and mutant proteins were diluted to a final concentration of *ca.* 10 μM. The samples were incubated at 4 °C in darkness in saturated oxygen solution. During this treatment, reduced FADH^–^ is converted to oxidised FAD, although spectral analyses revealed that the fraction of oxidised FAD differed among the different proteins. Thereafter, 10 mM 1,4-dithiotreitol were added to the protein solution. UV/vis spectra were recorded using a Jasco V550 photometer with temperature control adjusted to 10 °C. After the first recording, the sample was illuminated with blue light emitting diodes (*λ*_max_ = 470 nm) with a light intensity of 55 μmol m^–2^ s^–1^ at the position of the cuvette. Subsequent spectra were recorded at a series of time points as given in the results section. For data evaluation, complete spectra, 450 nm or 580 absorbance values, which stand for FAD in the oxidised or semireduced forms respectively, were presented.

#### Cofactor detection and repair assay

For detection of FAD and DMRL, 85 μM protein was denatured by 95 °C incubation for 5 min. The insoluble protein and the soluble chromophores were separated by 15 000 × *g* 10 min centrifugation and 10 μL supernatant were analysed by HPLC (Agilent system with a Gemini C18 column (50 × 4.60 mm, 110 Å, Phenomenex)). The HPLC buffer conditions were: 5% acetonitrile (ACN) in 0.1% formic acid for 0–5 min; 5–75% ACN in 0.1% formic acid for 5–25 min. The flow rate was set to 0.75 ml min^–1^ and the column temperature to 25 °C. Elution was monitored at 260 nm and 400 nm.

The photorepair reaction mixture contained 5 μM of the purified (6–4) photoproduct of t-repair_1 (Table S1†) and 8.5 μM protein in repair buffer (50 mM Tris–HCl, pH 7.0, 1 mM EDTA, 100 mM NaCl, 5 mM MnCl_2_, 5% (w/v) glycerol, 14 mM 1,4-dithiothreitol). Aliquots were irradiated with 400 nm light emitting diodes (250 μmol m^–2^ s^–1^) for 3 min. Thereafter, the reactions were stopped by heating to 95 °C for 10 min. Samples were centrifuged at 15 000 × *g* for 10 min and the supernatants analysed by HPLC (same column and system as above). The buffer conditions were: 7% acetonitrile (ACN) in 0.1 M triethylamine acetate (TEAA) (pH 7.0) for 0–5 min; 7–10% ACN in 0.1 M TEAA (pH 7.0) for 5–35 min. The flow rate was set to 0.75 ml min^–1^ and the column temperature to 25 °C.

### Computational methods

#### Model structures and molecular dynamics simulations

The structural model of PhrB WT has been derived from the X-ray crystal structure of Zhang *et al.* (PDB ID ; 4DJA).^17^ The Y391F, Y391A and Y391W mutants have not been crystallized. For the setup of the model structures, we suppose that the mutation of Tyr391 does not affect the structure of the remainder of the protein. Starting from the PhrB-WT model structure, we replaced the aromatic cycle of Tyr391 by a phenyl, a methyl or an indol ring. Two conformations of the indol ring are allowed by the protein structure, but steric hindrance prevents rotation from one to the other (see Fig. 5). In the first conformation, the Trp side chain can orient toward FAD being in a closer contact than in the second conformation. The conformations are termed Y391Wp (for proximal) and Y391Wd (for distal) in the following.

All mentioned simulations were performed with the GROMACS 5.0.4 package^34^,^35^ using the AMBER-SB99-ILDN force field.^36^,^37^ The force field parameters for neutral (oxidised) FAD and negatively charged cofactor FAD˙^–^ were taken from riboflavin and adenosine diphosphate (ADP) models developed in previous studies.^23^,^24^ The GAFF parameters^38^,^39^ were used for the DMRL antenna chromophore. The DMRL atomic charges were calculated by restrained fitting on the electrostatic potential (RESP)^40^,^41^ at HF/6-31G* (ref. 42 and 43) level with Gaussian 09 package.^44^ Bonded parameters of the cubic FeS-cluster were taken from ref. 45 and the charges were taken from ref. 46.

The loop region from residues 180 to 182, which might impact the DNA binding abilities,^17^ was not structurally resolved in the X-ray structure of WT PhrB. It was reconstructed using the MODELLER program.^47^ WT and mutated proteins (Y391F, Y391A and Y391W) were solvated in a 106.24 Å^3^ cubic box filled by TIP3P water molecules.^48^ Twelve sodium ions were added to create a neutral system.

Equilibration of the solvated proteins (WT and mutants) starts with a minimization step, followed by 100 ps MD in the NVT ensemble and 100 ps in the NPT ensemble. 100 ns of production NPT MD simulations were performed afterwards. Nose–Hoover thermostat^49^ was used to keep a constant temperature at 300 K and Parinello–Rahman barostat^50^ to keep the pressure at 1 atm. Covalent hydrogen bonds were fixed on a constant length by the use of the LINCS algorithm.^51^ The time step for the MD simulations was 2 fs.

#### Site energy and electronic coupling calculations

To treat the charge transfer processes, a quantum mechanical treatment of the active site has to be included *via* a so called combined Quantum Mechanics/Molecular Mechanics (QM/MM) scheme. The structural part of interest for charge transfer, which is treated at QM level, contains the side chains of amino acids involved in the called triad (**A**, **B** and **C**, see Fig. 2) and the isoalloxazine ring of FAD. The remaining atoms are treated classically using force fields (MM) and affect the QM zone by electrostatic interactions. Hydrogen link atoms^52^ are inserted at the QM/MM boundary, namely in the C_α_–C_β_ bond of **A**, **B** and **C** side chains or in the C1–C2 bond of the FAD D-ribitol tail.

To compute the electronic properties along the classical MD simulations, we use the semi-empirical Tight-Binding Density Functional theory (DFTB) method,^53^ which is derived from density functional theory (DFT) but roughly 2–3 orders of magnitude faster than standard GGA-DFT methods with medium sized basis sets. Running a fragmentation of the QM region into several functional parts speeds up the calculation significantly and allows to systematically correct for errors well known in DFT-GGA, like self-interaction error (for a detailed discussion see ref. 54 and 55). Each fragment is represented by only one frontier orbital and the chosen *i*-th fragment orbital (FO) of the fragment *m* is expressed in an atomic basis set *χ*_*μ*_ and determined by FO coefficients *c*_*μ*_^*i*^:^32^,^56^
1
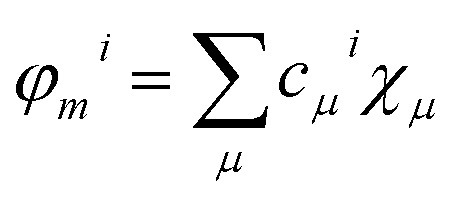



This FO-DFTB approach has been extensively evaluated and tested in previous publications^32^,^54^,^57^ and has been so far successfully applied to describe the charge transfer in photolyase,^23^ cryptochrome^24^ and DNA^58^,^59^ where the HOMO's of the fragments were used to calculate the evolution of electronic couplings and site energies.

The Hamiltonian *H*_*mn*_ matrix is built out of the FO coefficients and the Hamiltonian *H*_*μν*_ in the atomic-orbital-like basis function of the fragments:2
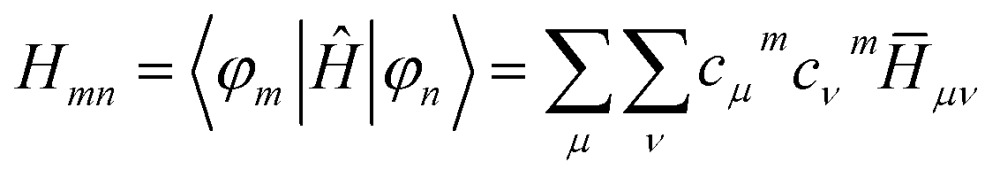



The diagonal elements of the Hamiltonian matrix correspond to the site energies *ε*_*m*_:

3 *ε*_*m*_ = *φ*_*m*_|*Ĥ*|*φ*_*m*_

The HOMO energies for different molecules show non-systematic errors, which can be corrected adding a constant energy shift depending on the chemical identity of the fragment. This correction was evaluated in ref. 58 for the relative energies of Trp and Tyr and expanded in this work to Phe and FAD relative to Trp (see Table S2†). The energy gap between two sites can be related to the driving force of a charge transfer as described in the Marcus theory.

The off-diagonal elements of the Hamiltonian correspond to the direct electronic coupling *H*_DA_ between two sites.

4 *H*_DA_ = *H*_*mn*_ = *φ*_*m*_|*Ĥ*|*φ*_*n*_

The FO-DFTB electronic coupling calculations were also validated in comparison with higher theoretical level results on set of organic stacked molecules.^56^,^60^

These matrix elements (*H*_DA_) can be used to compute two different charge transfer regimes. When large energy barriers occur along the charge transfer pathway, tunnelling matrix elements (*T*_DA_) can be computed (‘super-exchange tunnelling’), for small barriers, a ‘direct’ hopping-type mechanism may appear, which we treat using a QM/MM non-adiabatic MD technique, also based on the same computed matrix elements *H*_DA_.

#### Super-exchange tunnelling

In case of super-exchange tunnelling mechanism for a bridged charge transfer, the *n* fragments of the bridge B must be included in the electronic coupling *T*_DA_ calculation. The system is thus divided into the donor/acceptor (D/A) and bridge subspace and an effective Hamiltonian is calculated in which off-diagonal elements correspond to *T*_DA_ (for detailed discussions see ref. 54 and 61):5
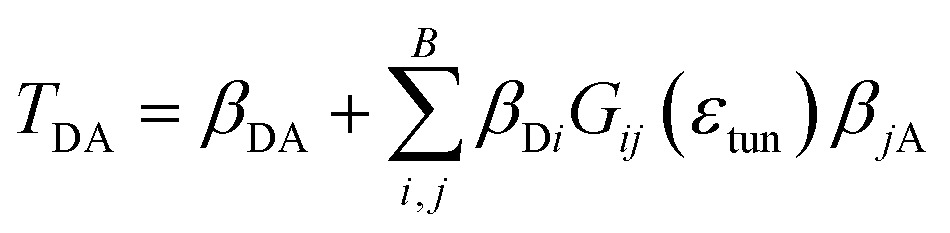
where *β*_DA_ describes the direct electronic interaction between D and A, *β*_D*i*_ and *β*_*j*A_ the electronic interactions between D or A and the *i* or *j*-th fragment of the bridge. The tunnelling energy *ε*_tun_ corresponds to the average of the eigenvalues of the effective Hamiltonian and *G*_*ij*_ is an element of the Green's function matrix, describing the probabilities for the electron to tunnel through the *i*–*j* space of the bridge.

#### Direct electron transfer

We established in our previous work a multi-scale method to describe charge transfer reactions in cryptochromes and photolyases.^23^,^24^ This non-adiabatic charge propagation scheme allows simulations where the charge transfer occurs on the same timescale as environment response charge state.

Nonadiabatic QM/MM MD simulations directly calculate the population of the charge which is located at a specific site of the QM zone.^62^ This method doesn't force a specific charge transfer process; however it can describe a hopping or even a band-like conduction.^32^,^57^ The wave-function associated with the transferred charge and atomic coordinates are simultaneously propagated solving time-dependent Schrödinger equation and classical Newton equations at every MD step, respectively. The excess charge corresponds to a second order perturbation of the neutral system Hamiltonian while environment interacts with the QM part as point charges. To take into account charge transfer, the partial charge of the QM part is calculated at every MD step to model the interaction of the moving charge with the charge distribution of the environment. More detailed description can be found in ref. 32, 57 and 62.

As for the electronic coupling calculations, charge propagation simulations were performed using an in-house GROMACS 4.6 version.^63^ The charge transfer between the triad and the isoalloxazine ring is initiated by the excitation of the FAD cofactor. The first transfer continuing the excitation was studied in some photolyases to happen within one picosecond.^64^ These two ultra-fast events were excluded in the previous studies^23^,^24^ to reduce complexity and to focus on charge transfer along the Trp triad. To keep consistency, the same exclusion was also applied here and the QM part consists in **A**, **B** and **C**.

Charge propagation simulations start with an electronic state where FAD˙^–^ is a radical anion and the first site **A** a radical cation. The hole on the first site is then propagated along the triad and moves in the reverse direction compared to the electron. These simulations were performed on PhrB WT and the Y391W mutant. Charge occupation of each site, varying from 0 (neutral state) to 1 (fully oxidized state) is followed during 1 ns QM/MM MD simulations. We randomly chose 20 to 25 starting structures from classical MD trajectories to guarantee well equilibrated systems while sampling different initial conditions. A kinetic model^23^,^24^ was used to fit the average occupations (from the individual simulations) of each site and thus determine the different reaction rate constants.

## Results

### Photoreduction of PhrB and mutants

**Fig. 3:**
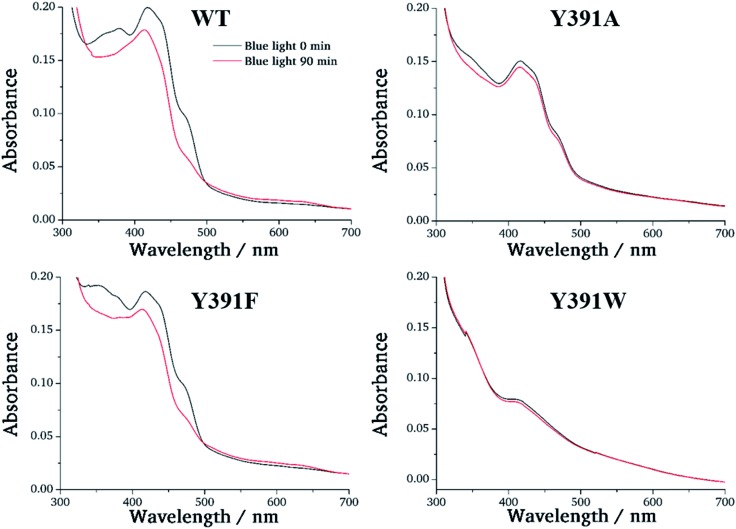
UV-visible spectra of PhrB WT and mutated proteins Y391A, Y391F and Y391W. Black line: without illumination; red line: illuminated by 470 nm blue light for 90 min.

For photoreduction studies, we generated the Y391F, Y391W and Y391A mutants of PhrB. All mutants and WT PhrB were expressed in *E. coli* and purified by Ni chromatography and size exclusion chromatography. Whereas protein yields of Y391F and Y391A are comparable to those of WT, the yield of Y391W is *ca.* 10 times lower. Absorbance spectra of Y391A and Y391F in the oxidised FAD state are comparable with WT (Fig. 3), although detailed analyses reveal different chromophore to protein ratios and/or different fractions of reduced FAD at starting time. The absorbance of the Y391W in the blue spectral range is very weak (Fig. 3). Chromophore analyses show that this mutant contains only (1.3 ± 0.1)% FAD and (1.8 ± 0.2)% DMRL as compared to WT. These values are (94 ± 1)% and (87 ± 3)% for FAD and DMRL of Y391F, respectively, and (53 ± 2)% and (53 ± 2)% for FAD and DMRL of Y391A, respectively. We propose that the replacement of Tyr 391, which is located close to FAD, by bulky Trp in Y391W results in opening of the FAD pocket and loss of FAD binding capacity. The DMRL pocket is formed by amino acids of the N-terminus and more distant from the mutation. The loss of DMRL results therefore probably from FAD depletion. The partial loss of both chromophores to equal percentages in the Y391A mutant supports this idea. The 410 nm peak in the spectrum of the Y391W mutant is assigned to the iron sulphur cluster.

**Fig. 4:**
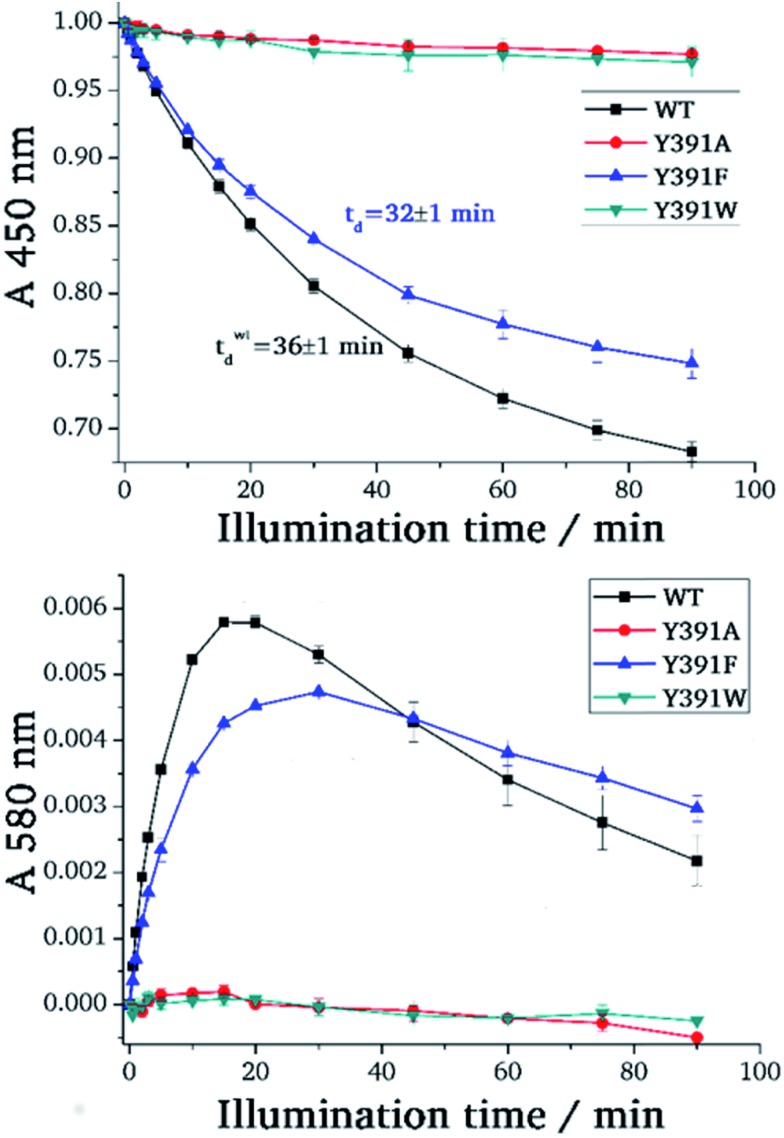
Photoreduction of PhrB and its mutants. Absorption values at 450 nm (upper picture) were taken from UV-visible spectra measured at indicated time points upon onset of blue-light illumination. For each protein, these values were normalized against the value measured at *t* = 0 min. Absorption values at 580 nm (lower picture) after subtraction of the *t* = 0 value and normalization to the absorbance at 280 nm.

During blue light irradiation, the spectra of WT PhrB and the Y391F mutant change in a characteristic manner. The transient increase at 580 nm, the maximum of the protonated FAD semiquinone, and the loss of absorbance at 450 nm, characteristic for the loss of oxidised FAD (Fig. 4), are comparable to data published earlier.^33^ Here, the relative *A*_450 nm_ decrease at *t* = 90 min in the Y391F mutant appears smaller than in WT. This can be due to slower photoreduction or smaller fraction of oxidised *vs.* total FAD in the mutant. Both decay curves can be fitted with monoexponential decay functions which yielded time constants of 36 ± 1 min and 32 ± 1 min for WT and Y391F, respectively. Thus, the rate of overall photoreduction is not affected by the Tyr to Phe replacement, but the oxidation state of Y391F was incomplete at the start of the photoreduction experiments. Formation and decay of the semiquinone intermediate absorbing at 580 nm is slower in Y391F (rise and decay times of 6 ± 0.3 min, 110 ± 30 min for WT and 7 ± 0.3 min, 200 ± 110 min for Y391F, respectively). This results shows that the role of Tyr or Phe differs in the first and second electron transfer. In summary, the present and published data^19^ show clearly that the replacement of Tyr by Phe into the proposed electron path does not block photoreduction. We do not observe any light induced absorbance changes in the Y391A mutant (Fig. 3). This result suggests that position 391 is critical for photoreduction, as proposed above. DNA repair in the presence of Mn^2+^ (ref. 11) is complete after 5 min for WT PhrB and Y391F mutant, whereas no repair activity is observed for Y391A and Y391W mutants under these conditions. When the repair time is prolonged to 120 min, Y391W repairs about (8.7 ± 0.7) % of damaged DNA, whereas with Y391A still no repair is observed.

### Molecular dynamics simulations

**Fig. 5:**
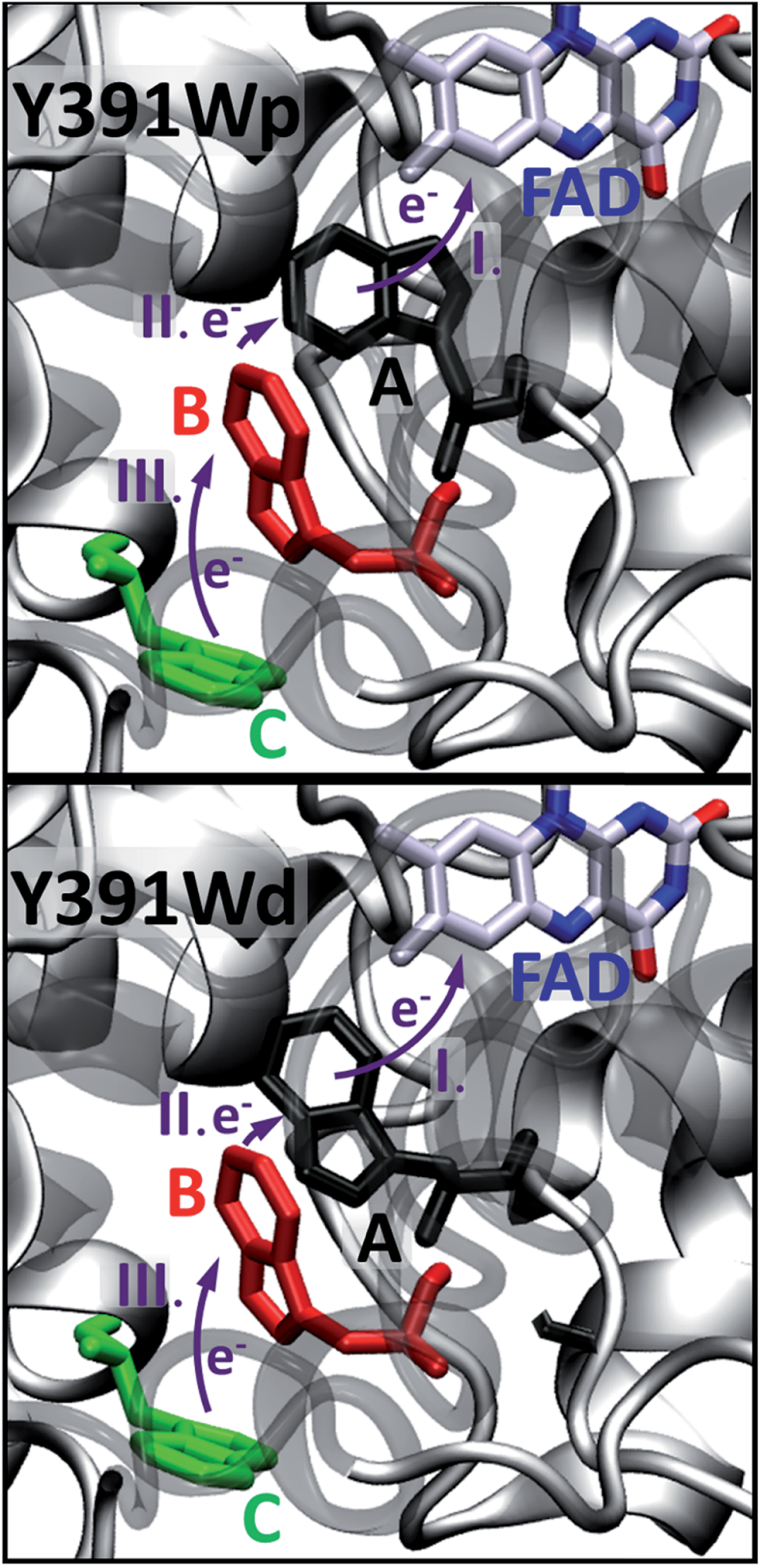
Two conformations, proximal (p, upper picture) or distal (d, lower picture) of **A** in the Y391W mutant.

We performed 100 ns classical MD simulations of PhrB WT and the Y391F, Y391A and Y391W mutant, based on the crystal structure of PhrB. In these simulations, no major conformational change of the overall protein structures is observed. The residues **A**, **B** and **C** (see Fig. 2) involved in the triad occupy similar positions in WT and the Y391F or Y391A mutants. In the model structure of the Y391A mutant, water molecules fill the space let by the replacement of Tyr to Ala (see Fig. S2†). In WT, Y391F and Y391A, a water molecule, present in the crystallographic structure, interacts with isoalloxazine O4 and **A** backbone (see Table S3 and Fig. S3†). The experimental findings for the Y391W mutant, which has lost both chromophores, suggests a more drastic impact on the protein structure which would require simulation protocols dedicated to protein folding and FAD docking. Our simulations, which follow the dynamics of Y391W on relatively short time-scales, however, allow us to investigate the theoretical role of an amino acid independent on large structural changes that might occur on much longer time-scales. Such simulations help us to compare the relationship between environment and the first site in different members of the cryptochrome–photolyase family. In the end, we have to combine experimental and theoretical results to obtain the maximum information for the role of each amino acid. The simulations for the Y391W mutant revealed two conformations of the Trp391 side chain (**A**) (see Fig. 5), denominated as Y391Wd and Y391Wp. In Y391Wd, **A** stays continuously close to the second Trp side chain **B** whereas in Y391Wp, **A** stays most of the time close and parallel to FAD isoalloxazine ring, but moves closer to **B** during a few nanoseconds (see Fig. 5, S4 and S5†).

**Fig. 6:**
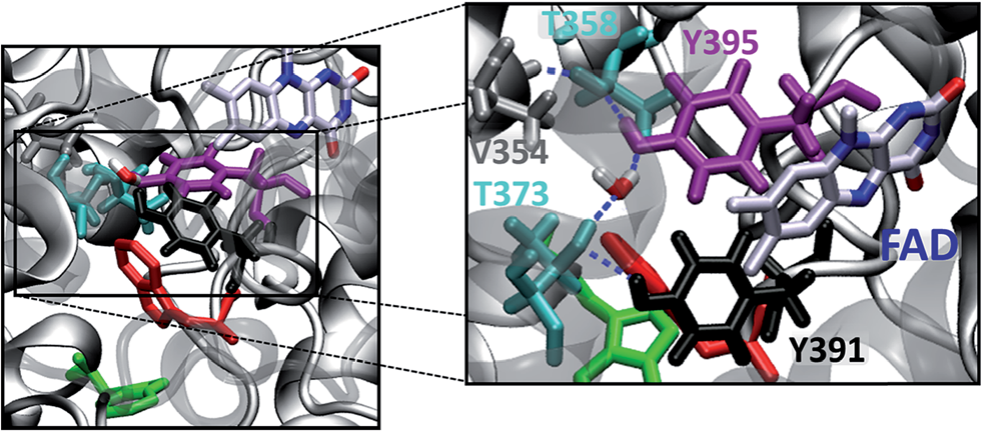
Active site with FAD and sites **A**, **B** and **C** (left). Hydrogen bond network involving the hydroxyl groups of Tyr391, Tyr395, Thr373, Tyr395, Thr358, the backbone carbonyl group of Val354 and a structurally characterized water molecule (right).

As shown in Table 1, the distances between all neighbouring sites (FAD–**A**, **A–B**, **B–C**) in WT and mutant structures are between 5 and 8 Å. These are typical nearest neighbour distances reported in our MD simulations of *Arabidopsis* cryptochrome and *E. coli* photolyase^22^–^24^ which allow for sufficiently large electronic couplings in order to enable fast charge transfer. The larger distances between FAD and the second neighbour between 12 and 13 Å (FAD–**B**) suggest that a direct electron transfer from **B** to FAD is unlikely because the electronic coupling decreases exponentially with distance in absence of charge transfer bridge. We also report the distance between FAD or **B** and another tyrosine, Tyr395 (see also Fig. 6), which is close to the charge transfer chain. Mutations have no impact on the position of Tyr395; it stays at around 8 Å from FAD and slightly less than 7 Å from **B**.

**Table 1:** Average distances (Å) between the centre of mass of the different aromatic sites (FAD isoalloxazine ring and Tyr, Phe or Trp side chain) involved in the FAD reduction in PhrB WT and its mutants Y391F, Y391A, Y391Wp and Y391Wd

	WT	Y391F	Y391A	Y391Wp	Y391Wd
FAD–**A**	7.89	8.21	—	7.19	7.54
**A–B**	5.29	5.24	—	6.09	5.45
**B–C**	6.65	6.51	6.51	6.22	6.40
FAD–**B**	12.28	12.70	12.36	12.76	12.07
FAD–Tyr395	8.28	8.06	8.29	8.03	7.67
Tyr395-B	6.77	6.93	6.95	7.01	7.00

### Site energy and electronic coupling

Upon excitation of FAD by light or by energy transfer from the excited antenna chromophore, this site is reduced to the negatively charged FAD˙^–^ species by an ET from the triad, which then in turn becomes positively charged. In a first step, we investigate the electronic structure of the neutral FAD–triad system by computing site energies and electronic couplings. In a second step, we compute the changes in the electronic structure due to the charge transfer. In previous work, we have studied *Arabidopsis* cryptochrome and *E. coli* photolyase in a similar way.^23^,^24^ Both proteins show a highly exergonic and fast charge transfer on a picosecond time scale. To investigate the effect of the mutation at site **A**, we compare the relevant parameters for charge transfer in the present study with these two reference systems.

The charge transfer parameters have been evaluated for oxidised FAD and a neutral triad along the 100 ns classical MD simulations on the neutral state of the protein using the FO-DFTB/MM scheme as discussed above. The orbital energies of **A**, **B** and **C** are called site energies and are a direct measure of the (relative) ionization potentials (IP) for the residue in the protein. The electronic couplings between the different partners are a measure for the charge transfer probability, *i.e.* they can be directly related to the prefactors in Marcus theory.^65^

**Fig. 7:**
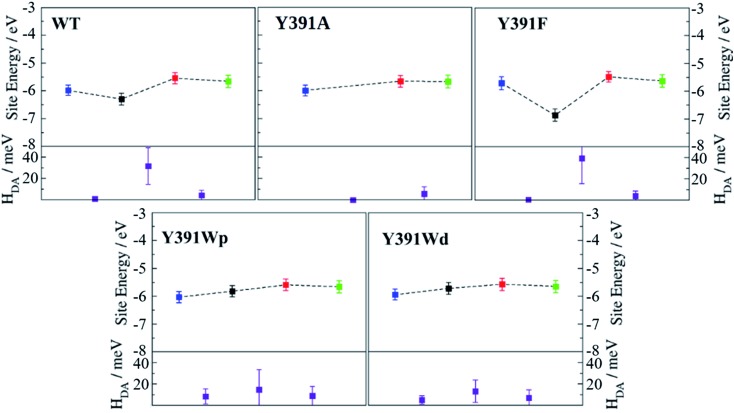
Average site energies (top, eV) and average electronic couplings (bottom, in meV) for PhrB WT and its mutants Y391F, Y391A and Y391Wp/d. Error bars denote the corresponding standard deviation. The different symbols in each site energies panel from left to right represent values for FAD (blue) and the residues **A** (black), **B** (red) and **C** (green). Electronic coupling *H*_DA_ symbols (purple) are positioned between the two sites considered as donor and acceptor.

Average values of energies and couplings are reported in Fig. 7, the error bars indicate the associated standard deviation. According to these data, site energies of Trp390 and Trp342 (**B** and **C**) are similar, independent of the chemical nature of **A**. The electronic coupling values associated with the charge transfer between these Trp's are about 4.1–4.2 meV for WT and the Y391F mutant, 5.9 meV for the Y391A mutant and between 7.0 and 9.0 meV for the two rotamers of the Y391W mutant. These values are comparable with those found in *Arabidopsis* cryptochrome and *E. coli* photolyase. The small increase of electronic coupling from WT to Y391W corresponds to a small decrease in **B–C** distance in the mutant (Table 1). For reference, we also show the location of the HOMO level of neutral FAD, which is the electron acceptor after excitation of one of its electrons.

The site energy of **A** as an aromatic residue (Trp, Tyr or Phe) inside the protein follows the same order as the HOMO energies in gas phase (Table 2, HOMO ≈ –IP): HOMO(Trp) > HOMO(Tyr) > HOMO(Phe). In the protein, **A** has a value of about –5.8 eV for Trp, which is substantially decreased to –6.3 eV for Tyr and to around –7 eV for Phe. In the Y391W mutant, the energy of **A** is similar to energies of **B** or **C**, leading to a small energy gap between the charge transfer partners (within about 0.3 eV). Electronic couplings are twice as large as in PhrB WT or Y391F. These results support the possibility of a charge migration involving a Trp triad in the Y391W mutant. The distal conformation Y391Wd facilitates **B** → **A** charge transfer by decreasing the **A–B** energy gap by 0.08 eV and the average **A–B** distance by 0.64 Å compared with Y391Wp (Table 1 and Fig. 7). On the contrary, in the proximal conformation, the aromatic ring of **A** and the isoalloxazine ring are in a close position which must enhance **A** → FAD charge transfer.

**Table 2:** Ionization potential (IP) determined in gas phase at ω97XD^66^/6-31g** (ref. 42 and 43) level for FAD and the different side chains considered in position 391

	FAD	Tyr	Trp	Phe
IP/eV	8.05	8.15	7.54	8.84

As expected, the lower site energies of Tyr in PhrB WT and Phe in Y391F may present a barrier for hole transfer from FAD, as clearly seen in Fig. 7. While our previous studies on *Arabidopsis* cryptochrome or *E. coli* photolyase showed a hopping type mechanism along the Trp-triad, one may expect in WT and Y391F a tunnelling mechanism to be in operation, which would lead to less efficient charge transfer. We calculated the electronic coupling between FAD and **B** which involves **A** as a bridge for the WT and Y391F by a previously published method.^54^ Our calculation shows a coupling of 0.06 and 0.02 meV for WT and Y391F which is ten-fold more than the direct (assuming no bridge residue) coupling between FAD and **B** (see ESI Table S4†). Electron tunnelling through the **A** side chain seems possible, as also indicated by the experiments on Y391F. Tunnelling involving a Phe and protein backbone has been also described for the *E. coli* photolyase.^67^ Therefore, in the case of Y391F, where a barrier of more than 1 eV is apparent, we definitely have to consider tunnelling, for the smaller barrier resulting from the presence of Tyr391 in WT this is not necessarily the case. This has been discussed for charge transfer in DNA,^68^ where small charge transfer barriers up to 0.4 eV may easily be overcome, especially for short bridges due to molecular fluctuations. The hole transfer between FAD and **A** falls in this range, allowing a direct hopping mechanism (Fig. 7). Electron hopping *via* Tyr would involve a transiently positively charged side chain, which must be followed by deprotonation. However, a nearby proton acceptor is missing in the PhrB structure, ruling out the deprotonation mechanism of Tyr391. Oxidised Tyr cannot be stabilised while the electronic coupling between **A** and **B** is strong (see Fig. 7). If FAD → **A** transfer occurs, the following hole transfer between **A** and **B** must be very fast.

Other charge transfer pathways can also be considered from the crystallographic structure. A charge transfer chain *via* Tyr399 and Tyr40 has been suggested in our previous experimental study.^19^ Spectral changes related to FAD reduction in Y399F mutant are slightly slower compared to WT. However, the absence of FAD reduction in both W390F and W342 mutants clearly indicates that Trp390 and Trp342 are essential in the charge transfer process, which cannot be compensated by a transport *via* Tyr399 and Tyr40. Consequently, the Tyr399–Tyr40 charge transfer pathway was rejected.

Another possibility could be a transfer between FAD and Trp390 *via* Tyr395 (see Fig. 6). This residue could substitute site **A** (Tyr391), since it has similar distances to FAD and site **B**, as shown in Table 1. Interestingly, theses distances do not change upon mutation at site **A**, *i.e.* the FAD binding pocket and the connection *via* Tyr391 is very similar in all variants. According to the computed electronic couplings and site energies (Table S5†), this pathway seems to be possible as well. However, our photoreduction studies for the Y391A mutant clearly indicate the absence of any charge transfer, *i.e.* the pathway does not seem to be a possible alternative. Although it has been shown that our scheme for the calculation of charge transfer couplings is very reliable for all couplings between the oxidisable residues,^60^ the couplings between FAD and site **A** respective Tyr391 are very small, *i.e.* a small change in geometry could lead to a different result. For example, the PhrB structure in solution (experimental photoreduction measurements) could differ from the crystal structure used for calculations. Further, the couplings are computed for neutral FAD in the ground state, whereas the active state is an excited state, which may have some impact on the value of the coupling. As a consequence, within the accuracy of the calculations reported here, the rather small value reported for the FAD–Tyr395 coupling (Table S5†) could as well turn out to vanish. Therefore, we believe that under the conditions the experiments are performed, the pathway *via* Tyr395 seems to be impeded. However, since an analogous alternative pathway has been reported for the PhrB homologue CryB,^18^ it could be interesting to investigate under which conditions this pathway could be activated in PhrB. This, however, would require further investigations which are beyond the scope of the present work. For the main focus of this work, it is very interesting to note that for this alternative pathway a tyrosine is the primary electron donor of FAD, *i.e.* highlighting the role of tyrosine in the charge transfer cascade, which is the main emphasise of this work.

To estimate the effect of a water molecule bridging the FAD and the backbone of site **A** (see ESI†), we also applied the pathways model from Beratan and co-workers.^69^ The couplings for these pathways are very small, therefore we did not consider these pathways further (see Table S4 and Fig. S6†).

**Fig. 8:**
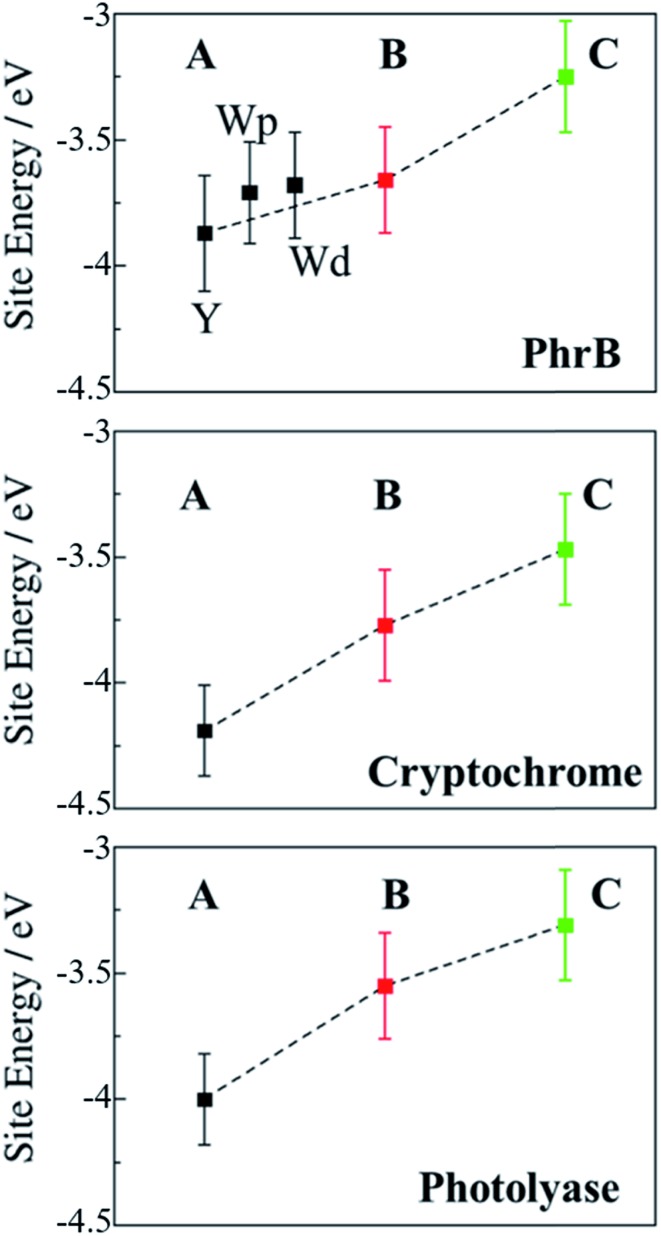
Average site energies (eV) of positively charged **A** (black), **B** (red) and **C** (green) for PhrB, *Arabidopsis* cryptochrome^24^ and *E. coli* photolyase.^23^ Error bars denote the corresponding standard deviation. For PhrB site **A**, the energies labelled with Y, Wp and Wd correspond to WT, Y391Wp and Y391Wd respectively.

We now discuss the energetics and couplings for the case, where FAD and one of the residues of the triad are charged. The energetic landscape is changed drastically when a charged FAD and a charged **A** are considered (Fig. 8). As discussed recently, the fast charge transfer in cryptochromes and photolyases can be explained by a steep downhill energetics, which results from the interaction of the charge with the protein and solvent environment.^23^,^24^ The first ET results in negatively charged FAD˙^–^ and a positively charged side chain on one site of the triad. Charge separation has a sizable effect on the site energies. The positive charge on **A**, **B** or **C** leads to a strong polarization of the environment. This polarized environment in turn leads to a stabilization of the charge at the respective site. In Marcus theory, this effect is called outer-sphere reorganization energy; in our simulations it manifests itself by the lowering of the site energies with regard to the neutral states. For each charge state on **A**, **B** and **C**, we compute the site energies along 1 ns MD simulations containing the charge on the respective site during the MD simulations, as shown in Fig. 8. We have discussed this effect in some details in our previous work, showing that the solvent has a distinct impact, in particular on those sites which are more solvent exposed,^24^ like site **C** which is located on the protein surface.

In previously studied *Arabidopsis* cryptochrome or *E. coli* photolyase, the positive charge stabilization follows a downhill scheme, with an increasing energy gap between neutral and charge residue from **A** to **C** and where neighbouring sites have an energy difference of about 0.5 eV (Fig. 8). This leads to the fast charge transfer in a picosecond regime.

In PhrB, a similar stabilization on **C** occurs, this results from the solvent exposure. However, surprisingly also site **A** is massively stabilized, for Tyr even more than for both Trp rotamers. In the latter, **A** and **B** site energies are similar and the charge transfer from **B** to **A** no longer follows the downhill scheme described for the WT, *E. coli* photolyase or *Arabisopsis* cryptochrome. In total, the energy difference between site **C** and **A** in Y391W is only half of the value compared to the other two systems (Fig. 8), which has a drastic effect on the charge transfer equilibrium.

**Fig. 9:**
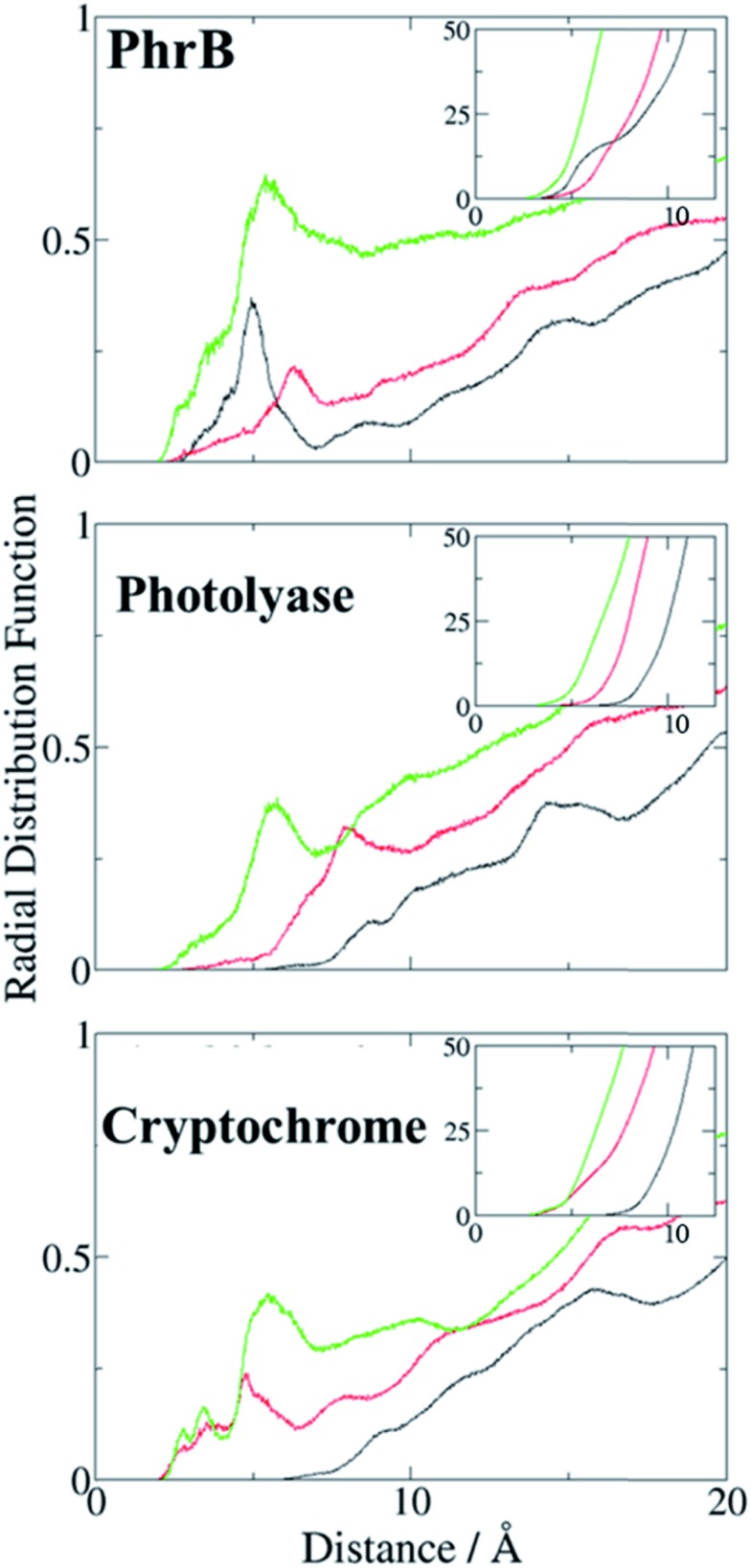
Radial distribution function of water molecules around **A** (black), **B** (red) and **C** (green) in PhrB WT, *E. coli* photolyase, *Arabidopsis* cryptochrome. Integration of the radial distribution curves is given in insets.

There is an obvious energetic difference for the site **A** Trp in Y391W, *E. coli* photolyase and *Arabidopsis* cryptochrome. In Y391W, **A** is nearly isoenergetic to **B**, while in the other proteins, positively charged **B** is more stable than **A** by about 0.4 eV. In our previous work, we have analysed structural reasons for this energy gap between **A** and **B**. In the *E. coli* photolyase or *Arabidopsis* cryptochrome, **A** is buried in a pocket with more than 5 Å distance from any water molecule, as documented by calculation of water distribution functions along MD simulations (Fig. 9). On the contrary, water molecules can easily move toward **A** in PhrB: the radial distribution function of water around Tyr391 presents a peak around 5 Å, while the first peak around **B** is observed at 7 Å (Fig. 9). Moreover, a stable hydrogen bond network, involving Thr373, a water molecule, Tyr385, Thr358 and the backbone of Val354 (Fig. 6) can also participate in **A˙^+^** stabilization in our simulation. In *E. coli* photolyase and *Arabidopsis* cryptochrome, sites **B** and **C** are stabilised by solvent interactions, leading to a larger solvent reorganization energy. This explains the downhill energetics as shown in Fig. 8. On the contrary, water molecules close to the FAD–**A** complex in PhrB help to stabilize the charge-separated RP-**A** (FAD˙^–^– **A˙^+^**) state, and compensate the unfavourable intrinsic IP of Tyr. Likewise, this could impact the charge transfer efficiency in the Y391W mutant by increasing the probability to localize the charge on the first member of the triad.

### Charge transfer simulations

**Fig. 10:**
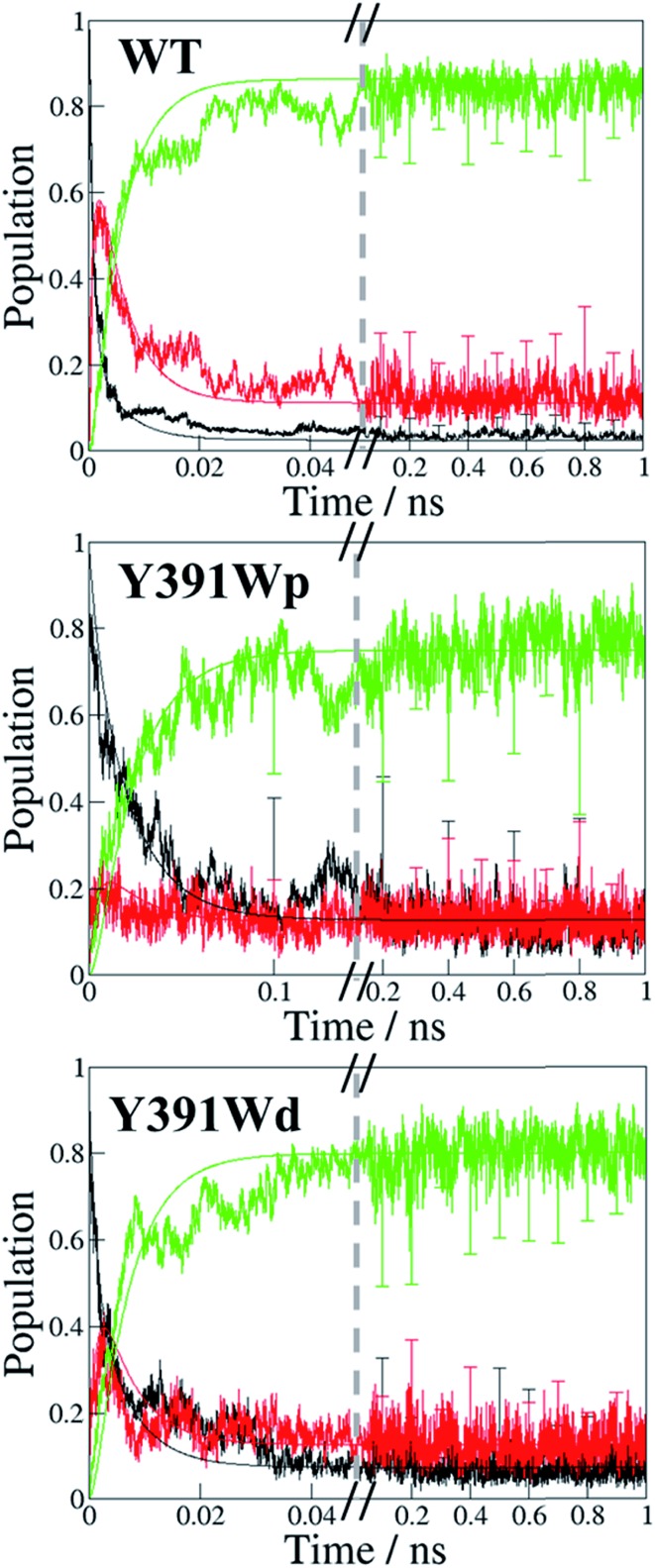
Time dependent evolution of the charge occupation along the triad in PhrB WT, Y391Wp mutant and Y391Wd mutant. Black line: RP-**A**; red line, RP-**B**: green line: RP-**C**. The graphs show the average occupation from all simulations of 1 ns length for WT or Y391W mutants respectively (see Fig. S7† for more detailed results). Error bars denote the standard deviation each 0.1 ns. The curves resulting from a fit using a kinetic model^24^ as described in the text are also shown using the same colours.

To study the implication of the energetic landscape on the charge transfer dynamics, we performed unbiased simulations of the charge transfer through the residues **A**, **B** and **C** in WT protein and Y391W rotamers. Y391F and Y391A are not considered in this part as Phe and Ala cannot be oxidised. These simulations indicate the formation and lifetime of each radical pair state: RP-**A** (FAD˙^–^–**A˙^+^**), RP-**B** (FAD˙^–^–**B˙^+^**), RP-**C** (FAD˙^–^–**C˙^+^**), summarized in Fig. 10. We present the averages over 25 simulations for WT and 20 simulations for the two Y391W mutants; more details and charge transfers movies are given in ESI.†

The charge is considered to be on a specific site when the occupation of the site is larger than 50%. The time dependence of the average site occupations have been fitted using a kinetic model previously described^23^,^24^ which allows to obtain rate constants (Table 3) corresponding to the following steps:6
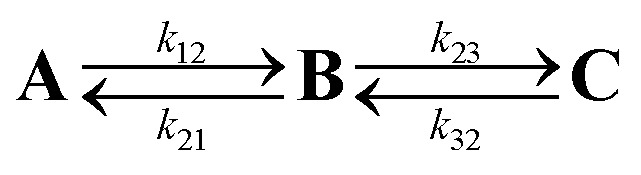



**Table 3:** Hole transfer rate constants (ns^–1^) described in eqn (6) for PhrB WT, Y391W mutant rotamers, *E. coli* photolyase (PL) and *Arabidopsis* cryptochrome (CRY). We used a polarization parameter of 1.4 (ref. 23)

	PhrB WT	Y391Wp	Y391Wd	*E. coli* PL^23^	*Arabidopsis* CRY^24^
*k*_12_	870	63	237	88	190
*k*_21_	193	66	256	87	85
*k*_23_	199	124	231	64	70
*k*_32_	25	20	36	6	10

In WT, the population of the first radical state RP-**A** drops within a few ps and no back transfer is observed. The second state, RP-**B**, is transiently occupied by 60% of the total positive charge before decay and formation of the third state RP-**C**. After 25 ps simulation time, the charge distribution remains stable, with roughly 85% of the charge on the third state, while 15% remains on the second state. Due to the averaging over several simulations, these numbers show a statistical distribution rather than a charge delocalization over different sites. The positive charge is therefore well stabilized on the solvent accessible Trp342. No backward charge transfers from **B** to **A** occur during 1 ns of simulation time. The very fast first charge transfer from Tyr391 to Trp390 shows a significantly larger transfer rate than that calculated for plant cryptochrome.^1^ PhrB *k*_23_ and *k*_32_ are consistent with values from *Arabidopsis* cryptochrome and *E. coli* photolyase, with a backward transfer 10 fold smaller than the forward one.

The Y391W mutants show a different behaviour. On average, the hole remains on RP-**A** during the first 50 ps in Y391Wp and during the first 21 ps in Y391Wd, respectively. The final stabilization of 70–80% of the charge on site **C** occurs after 70 ps for Y391Wp and 33 ps for Y391Wd. For the **A–B** transfer, the forward and backward rate constants are very similar in each rotamer simulation, as shown in Table 3. In Y391Wd, the *k*_21_ rate is also close to *k*_23_. For the transfer from **B** to **C**, the forward rate is 10-fold higher than the backward rate. All rate constants are in the same order of magnitude as those of *Arabidopsis* cryptochrome or *E. coli* photolyase. The main difference between the Y391W mutant and *Arabidopsis* cryptochrome is the strong back transfer rate from **B** to **A** (*k*_21_ in Table 3).

Indeed, during all charge transfer simulations of our different cryptochromes and photolyases proteins, we observe several backward transfers to **A**. No backward transfer is present in PhrB WT simulations, as the first residue is a Tyr. We compare the number and the stability of backward transfers in Table 4 for different systems: Y391Wp/d and *E. coli* photolyase. In Y391W, we observe about 30 crossings in which the positive charge moves back to **A** and forms a stable RP-**A** for at least 500 fs which is ten-fold more than in *E. coli* photolyase. The difference between the two conformations of Y391W is related to the **A–B** distance: in Y391Wd, **A** is closer to **B**, which facilitates backward and forward charge transfer between them. On the contrary, in Y391Wp, **A** is closer to negatively charged FAD which contributes to stabilize the positive charge on **A**. Furthermore, a charge recombination on the isoalloxazine ring becomes more likely. Nevertheless, in 100 ns MD simulation of Y391Wp, motion of **A** to a distal conformation is observed and can also contribute to an enhanced charge transfer between the two Trp residues. In both conformations, the charge is more often back transferred to **A**, but stays less time for one transfer than in *E. coli* photolyase.

**Table 4:** Average number *ν* of backward transfer from **B** to **A** and average total time *τ* of occupied **A**. The charge is considered to be on **A** when the occupation of the site is larger than 50%. **A** transfer is counted in *τ* when the charge stays more than 500 fs on site **A**. 20 charge transfer simulations of 1 ns are taken into account for Y391Wp/d and 22 for *E. coli* photolyase respectively

	Y391Wp	Y391Wd	*E. coli* PL^23^
*ν*	29.8	35.2	3.5
*τ* (ps)	92.0	22.5	67.5

## Discussion and conclusion

In most members of the cryptochrome–photolyase family, the central ET pathway contains a triad of Trp residues, from the surface of the protein to the FAD chromophore. The mechanism commonly accepted for the FAD photoreduction consists of three successive hole transfer steps: FAD* → **A**, **A** → **B** and **B** → **C**.^31^

Like other members of the cryptochrome–photolyase family, PhrB is able to reduce FAD upon light absorption *via* a long range ET involving aromatic residues. Site directed mutagenesis experiments have shown that two Trp, Trp390 and Trp342, are essential for the reduction process. The distance between the isoalloxazine ring and Trp390 is roughly 12–13 Å (Table 1) and thus too far from FAD for direct ET. There is no other Trp in the structure that can complete the triad, which raises the question of the role of the closest residue to FAD involved in charge transfer.

The presence of Tyr391 is intriguing: it is situated between FAD and Trp390 in a suitable place to take part in the ET, but its oxidation appears – at first sight – not required for FAD reduction: (i) mutation of Tyr391 to redox inert Phe residue neither blocks FAD photoreduction nor DNA repair; (ii) in 464 PhrB homologs, this residue is either Tyr or Phe (Fig. S1†). However, experimental mutation of Tyr391 to Ala blocks FAD photoreduction and DNA repair, underlining its relevance in electron transfer process. This observation rules out other alternative pathways *e.g.* through Tyr395 or water molecules.

In both WT and Y391F mutant we find an energy barrier at site **A**. Therefore, we considered a super-exchange tunnelling process for both residues, which is a viable hypothesis to explain the experimental charge transfer between FAD and Trp390 in Y391F mutant where a large energy barrier occurs, but the computed couplings are sufficient to allow charge transfer according to this mechanism.^70^ We observe similar electronic couplings between FAD and **B** when a Tyr or Phe aromatic cycle is included as a bridge. One can notice that in WT and Y391F, the Tyr391 or Phe391 aromatic cycle is parallel to the isoalloxazine ring of FAD and in a suitable conformation for π–π orbitals interaction.

Hole transfer *via* tyrosines is usually not favourable due to the high ionisation potential, which is 0.6 eV larger than for tryptophans (Table 2). The oxidised state of tyrosines can be stabilized by proton transfer, but there is no proton acceptor in the neighbourhood of Tyr391. Therefore, an efficient hopping mechanism, where Tyr391 is transiently oxidized, seems improbable at first sight. The energy of the Tyr391 HOMO, however, is significantly reduced in PhrB. The Tyr391 oxidized state is stabilized by the protein environment instead, in the immediate environment a hydrogen-bond network (Fig. 6) can attract the proton from Tyr391 and allow the O–H bond elongation to compensate an electronic density decrease on the cycle. Therefore, the charge transfer from FAD to **B***via* a transiently oxidized tyrosine seems to be possible. In addition to tunnelling, a much more efficient hopping regime seems to be enabled in PhrB due to the specific protein environment of site **A**. Mutation of Tyr391 to Phe disables this efficient pathway, but does not impede tunnelling as shown by both, experimental and theoretical results. Mutation to Ala, however, blocks the pathway, as shown in experiments and calculations, and thereby blocks FAD photoreduction due to vanishing electronic couplings.

Most photolyases and cryptochromes carry a Trp triad. In these cases, the strong exothermicity of the charge transfer results from stabilization of the positive charge by the solvent, as discussed in detail in our previous work.^22^–^24^ The question arises therefore, why PhrB makes use of a Tyr at site **A** instead of a Trp residue.

**Fig. 11:**
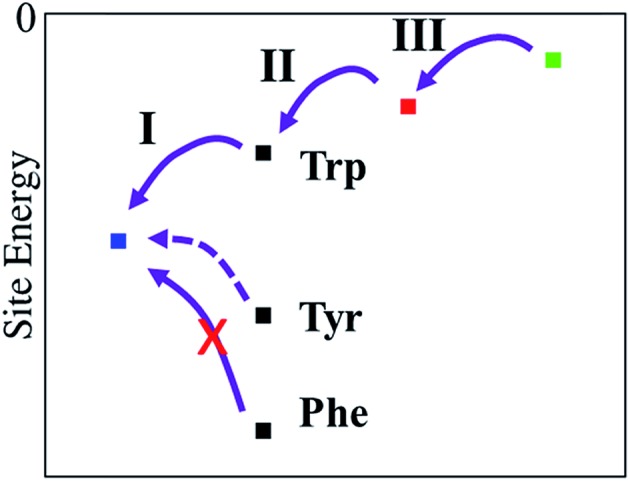
Relative site energies (from left to right) of FAD, site **A**, site **B** and site **C**. For site **A**, three residue types have been investigated, Trp, Tyr and Phe.

Electron transfer can be described by an energy landscape as shown schematically in Fig. 11. It is clear that a Tyr substitution would introduce significant barriers into the charge transfer pathway when it would be placed into the photoreduction pathway of *e.g. E. coli* photolyase. The barrier, when estimated using the gas-phase ionisation potentials (Table 2) is in the order of 0.6 eV, which surely would block an efficient hopping type charge transfer, as found for proteins with a Trp-triad. In PhrB, however, both Tyr and Trp at site **A** show a stronger stabilization, due to interactions with water and the protein environment, when compared to other members of the cryptochrome–photolyase family which we have already simulated.^23^,^24^ Since site **A** in PhrB is very close to water molecules in the binding pocket, the intrinsic IP of Tyr and Trp are substantially stabilized by this polar environment. This stabilization results in a downhill hole transfer from FAD to **C** in PhrB WT but in similar energies for site **A** and **B** in Y391W mutant. The calculations suggest a reason for the presence of Tyr instead of Trp in PhrB. The forward transfer is less efficient for Tyr compared to Trp due to the slightly higher IP, however, the back-transfer seems to be too efficient in Y391W mutant, which may lead to unproductive cycles. The Y391W mutation, because of the higher intrinsic IP of Trp, disrupts the downhill energetics and allows charge recombination of FAD. The protein may therefore trade a slightly less efficient forward transfer for blocking back-transfer when using a tyrosine at site **A**.

Absorbance spectra showed that the Y391W mutant has lost both FAD and the antenna chromophore DMRL almost completely. The loss of FAD could be due to a modification of the chromophore pocket by Trp, which is larger than Tyr. The small percentage of DNA repair by the Y391W mutant suggests that a small fraction is still able to bind FAD. Quantitative comparisons between photoreduction of Y391W and WT are not possible experimentally. However, our simulations of the Y391W mutant (where loss of FAD was not considered) provide valuable insight of the importance of the Tyr residue in comparison with previously studied cryptochromes and photolyases.^23^,^24^ The radical pair state of RP-**A** is stabilized in our Y391W simulations due to strong electronic couplings and small energy gaps between Trp390 and Trp391 and charge is transferred to Trp342 within 33–70 ps (Fig. 10). Although rate constants for the hopping mechanisms in the Y391W mutant of PhrB are comparable with the Trp triads in *E. coli* photolyase and *Arabidopsis* cryptochrome,^23^,^24^ backward charge transfers to **A** occurs more frequently in the PhrB mutant than in these two proteins. Such back transfer increases the risk of charge recombination on FAD and hence a more likely inefficient charge transfer mechanism. If a Trp residue corresponded to the first site and if the FAD binding were efficient, the environment would stabilize the RP-**A** state in PhrB obviously more than in Trp triads of other members of the cryptochrome–photolyase family.

Taken together, our experimental and theoretical results indicate the following: the protein environment is quite different in PhrB from other groups of photolyases and cryptochromes. Residues bigger than Tyr at position **A** result in loss of FAD binding. Solvent can come closer to **A**, stabilizing the FAD˙^–^–**A˙^+^** state due to reorganization energy. The presence of a Tyr residue instead of a Trp at this site preserves the structure, the energetics, and therefore the function of PhrB.

## Conflicts of interest

There are no conflicts to declare.

## Supplementary Material

Supplementary information

Supplementary movie

Supplementary movie
