# Supplementary material for: Functional role of an unusual tyrosine residue in the electron transfer chain of a prokaryotic (6–4) photolyase
Source: Chem Sci. 2017 Dec 11;9(5):1259–72. doi: 10.1039/c7sc03386a (PMC5887102; doi:10.1039/c7sc03386a)
Supplement: Supplementary file 1 [file SC-009-C7SC03386A-s001.pdf]

Electronic Supplementary Material (ESI) for Chemical Science.
This journal is © The Royal Society of Chemistry 2017

Functional Role of an Unusual Tyrosine Residue in the Electron Transfer Chain of a Prokaryotic (6-4) Photolyase

Daniel Holub,[1] Hongju Ma,[2] Norbert Krauß, [2] Tilman Lamparter,[2] Marcus Elstner,[1] Natacha Gillet[1]

1. Department for Theoretical Chemical Biology, Institute for Physical Chemistry, Karlsruhe Institute for Technology, Kaiserstr. 12, 76131, Karlsruhe, Germany
2. Botanical Institute, Karlsruhe Institute for Technology, Fritz Haber Weg 4, 76131, Karlsruhe, Germany

email: natacha.gillet@kit.edu

Conserved residues in FeS-BCP members

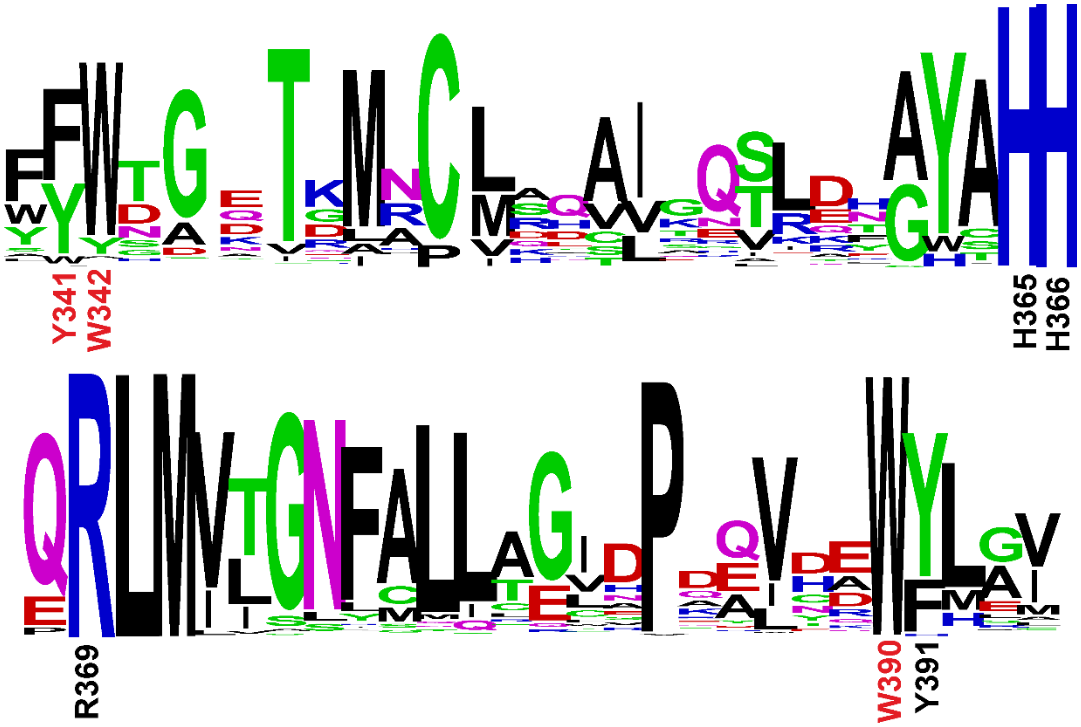

Figure S1: Sequence alignment of the 464 PhrB homologs using WebLogo web based application.[1,2]

Oligonucleotide sequence for DNA repair

**T-REPAIR_1**	**AGGTTGGC**
**Y391W FW**	CGGTGCATCGGTGG**TGG**CTCGAGGTCTATGCG
**Y391W REV**	CGCATAGACCTCGAG**CCA**CCACCGATGCACCG
**Y391A FW**	CGGTGCATCGGTGG**GCG**CTCGAGGTCTATGCG
**Y391A REV**	CGCATAGACCTCGAG**CGC**CCACCGATGCACCG
**Y391F FW**	GCGGTGCATCGGTGG**TTT**CTCGAGGTCTATGCG
**Y391F REV**	CGCATAGACCTCGAG**AAA**CCACCGATGCACCGC

Table S1: Oligonucleotide sequences for DNA repair studies (first line) and site-directed mutagenesis (2nd to 7th line). The TT pair that yields the (6–4) photoproduct is printed in bold. The triplets of the mutation sites are underlined.

Parameterization of DFTB-FO energies

In order to describe the hole transfer energetics correctly, the DFTB HOMO energies must be corrected compared to ionization potential (IP) calculated at DFT level (ωB97XD[3]/ 6-311g**[4,5]). The relative energies of Trp and Tyr have been determined in a previous work.[6] We use the same protocol for relative energies of Trp and Phe. In each molecule, the DFT IP and the DFTB HOMO energy of the neutral state are considered to determine the correction which will be added to the site energy. We calculate the energy difference between the IP of two sites at DFT level and compare this difference to HOMO energy of DFTB. The obtained value (Table S1) is added to DFTB HOMO energy to reproduce the DFT difference at DFTB level.

FAD requires a more specific treatment because of the excitation step needing for hole transfer. The interacting orbitals should be the HOMO-1 of FAD* and HOMO of **A**. We can consider that the HOMO-1 of FAD* correspond to the HOMO of FAD semi-occupied following different approximations:

- we neglect the impact of the occupation of the LUMO of FAD on the HOMO energy
- we neglect the relaxation of the environment induced by modification of the dipolar moment of FAD after excitation. The hole transfer from FAD* to **A** is supposed to occur very fast (< 1 ps), the environment has no time to relax before hole transfer.

We thus compare the DFTB HOMO energy with the DFT IP of FAD to determine the shift added to DFTB HOMO energy.

	Trp	FAD	Tyr	Phe
**IP / eV**	7.54	8.05	8.19	8.84
**HOMO / eV**	5.32	5.63	5.64	6.41
**Shift / eV**	0	0.20	0.33	0.21

Table S2: DFT ionization potential and DFT HOMO energies. Shift values correspond to the difference between IP and HOMO energy gap and are added to DFTB HOMO

Water molecules between FAD and A backbone

In the PhrB crystallographic structure, a water molecule interacts with FAD O4 or N5 and **A** backbone.[7] This position is conserved all along the MD simulations of WT and Y391F (Table S3). In Y391A, the available space between FAD and Trp390 is filled up with 4-5 water molecules (Figure S2). The water bridge between FAD and **A** backbone is maintained during MD as five water molecules occupy successively this position during the 100 ns Y391A simulation (Figure S3).

	O4-water	N5-water	water-O_{bb}
**WT**	2.92±0.26	3.17±0.22	3.00±0.40
**Y391F**	3.29±0.32	3.41±0.58	3.48±0.72

Table S3: Average distances (Å) between the centre of mass of the crystallographic water molecule and O4, N5 of FAD or between the centre of mass of the crystallographic water molecule and the oxygen atom of A backbone O_{bb}.

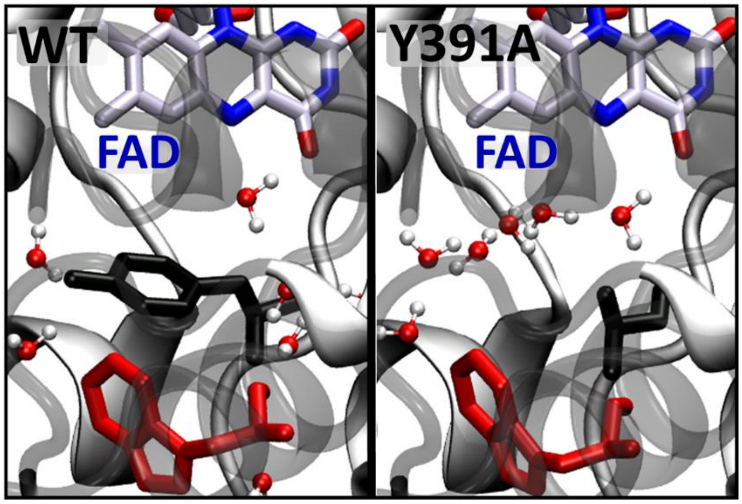

Figure S2 : comparison between FAD-A neighbouring in WT(left) and Y391A (right).

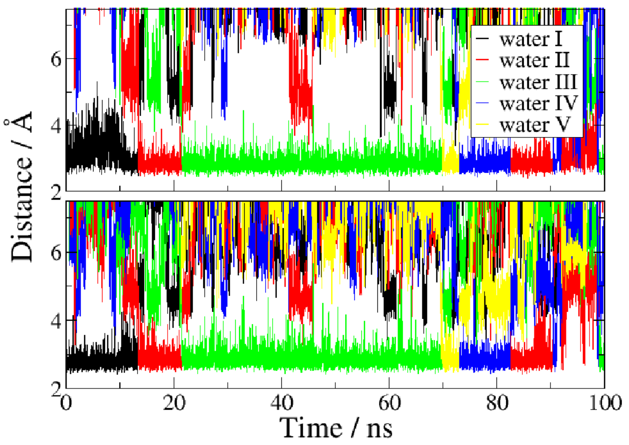

Figure S3: Distance between the centre of mass of successive water molecules and FAD N5 (top) or oxygen atom of A backbone (bottom).

Interaction between FAD and A in Y391W rotamers

We performed 100 ns simulations for four different systems (WT, Y391F, Y391Wp and Y391Wd). Each simulation shows a stable protein and a small RMSD fluctuation of the mutated **A** with the exception of a short turn of the W391 in Y391Wp (Figure S4). This movement happens between the 24 ns and 35 ns and the distance between FAD and Trp391 is increased by around 2 Å. In the proximal position of Trp391, the aromatic planes of **A** and FAD are parallel and the N5 and indol nitrogen atom are quite close (around 4 Å) but no hydrogen bond between them is observed (Figure S5). Movement of Trp391 has a small impact on isoalloxazine ring RMSD.

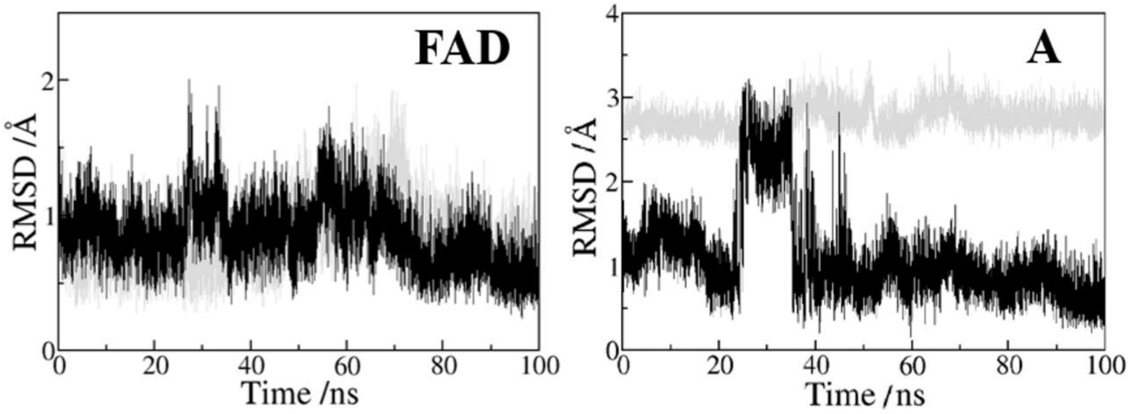

Figure S4: RMSD evolution of FAD (left) and A (right) along Y391Wp (black) and Y391Wd (grey) simulations (compared to final position of Y391Wp). The highest RMSD values for Y391Wp correspond to the position of A closest to B, similar to Y391Wd position.

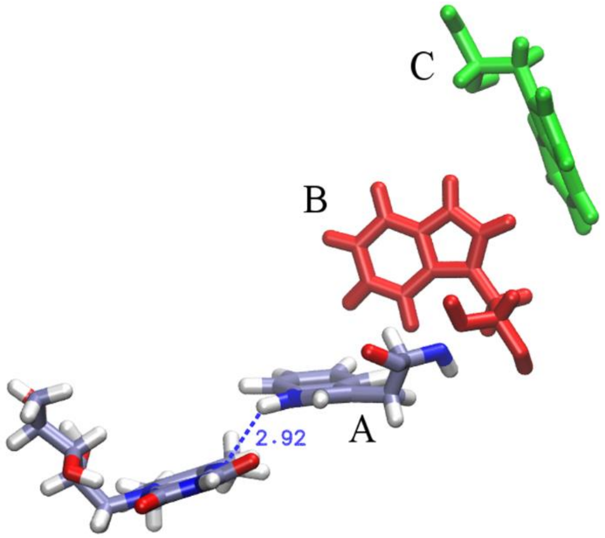

Figure S5: proximal position of A in the Y391W mutant: A aromatic plane is parallel and perpendicular to FAD and B aromatic planes respectively.

Superexchange tunneling through tyrosine and phenylalanine

We calculated the superexchange tunneling with the same method as published in ref [8,9]. We used the HOMO of FAD as donor, **A** (Tyr or Phe) as bridge and **B** (Trp390) as acceptor for an electron hole. We also used the pathways plugin in VMD[10,11] to perform a Pathways model analysis[12] taking into account **A** (Tyr, Phe or Ala) and water molecule as bridge.

The different pathways are given in Figure S6 for WT and Y391F. The pathways involving water molecules represent 80%, 75% and 88% of the strongest pathways in WT, Y391F and Y391A simulations respectively. The pathways through **A** aromatic cycle and associated electronic coupling damping values have been obtained deleting the water from the bridge. All average electronic coupling damping values are reported in Table S4.

	**WT**	**Y391F**	**Y391A**
**H_{DA} FAD-B / meV**	0.003	0.005	0
**T_{DA} FO-DFTB / meV**	0.062	0.018	-
**T_{DA} Pathways Relative values (x10^{-3})**	0.028	0.013	-
**T_{DA} Pathways H_2O Relative values (x10^{-3})**	0.835	0.255	0.986

Table S4: Average direct H_{DA} and bridged T_{DA} electronic couplings obtained from FO-DFTB calculations or pathways model with and without the water molecule in the bridge. The pathway model only computes relative values with respect to a reference, i.e. the damping of the charge transfer couplings through covalent bonds, hydrogen bonds and through space.

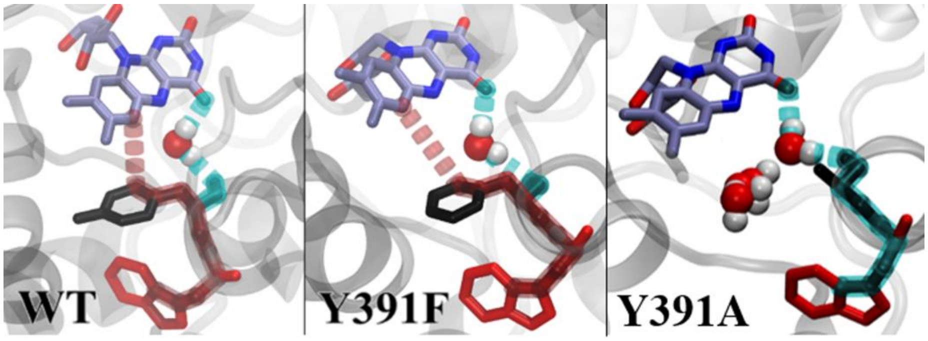

Figure S6: Two possible tunnelling pathways from B to FAD involving A aromatic cycle (red) or A backbone and a water molecule (cyan) in WT, Y391F and Y391A.

Charge transfer through Tyr395

Because of its proximity to the charge transfer chain, Tyr395 can be considered in a secondary charge transfer pathway also comprising Trp390 and Trp342. We consequently calculated the site energy of Tyr395 and its electronic coupling with both FAD and **B** in WT, Y391F and Y391A mutant (Table S5). The electronic coupling between FAD and **B** involving Tyr 395 as a bridge has been also determined at FO-DFTB level.

	E(Y395) /eV	H_{DA} FAD-Y395 / meV	H_{DA} Y395-B / meV	T_{DA} FAD-B /meV
**WT**	6.19	0.173	17.2	0.020
**Y391F**	6.26	0.168	10.7	0.007
**Y391A**	6.11	0.115	11.0	0.015

Table S5: Site energy E of Tyr395, and electronic couplings associated to charge transfer pathways including Tyr395 as a charged intermediate (H_{DA}) or as a bridge for tunnelling between FAD and B (T_{DA}).

Detail of the Direct Simulations of charge transfer

We performed individual charge transfer simulations for the WT, Y391Wp and Y391Wd to calculate the reaction rates. Structures randomly selected out of the 100 ns MD trajectory were used as starting structures (25 × WT, 20 × Y391Wp and 20 × Y391Wd). Population on each site for the different MD simulations are given in Figure S4. Films (also given in SI) show the charge propagation on one simulation of 500 ns of WT or Y391Wp. In this simulation, residues are coloured according to their charge: blue for positive charge, red for neutral and green for intermediate charge.

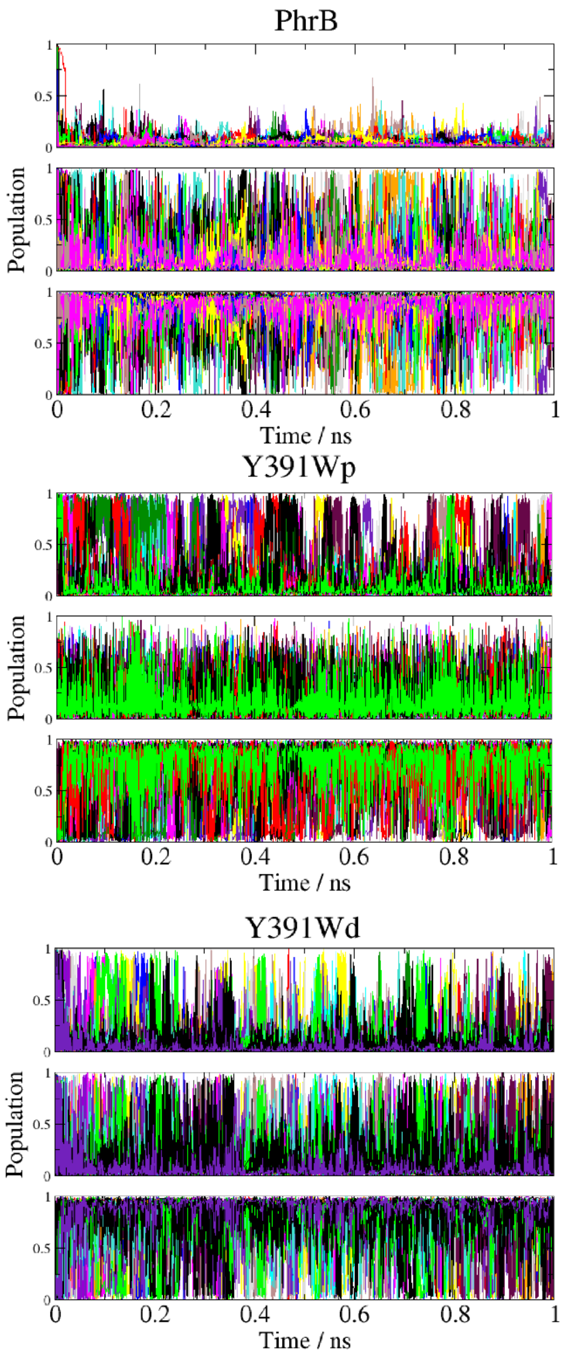

Figure S7: Evolution of the population of (from top to bottom for each system) A, B and C in WT, Y391Wp and Y391Wd during the charge propagation simulations. At time 0, A is positively charged (population = 1) while B and C are neutral (population = 0).

References

1 T. D. Schneider and R. M. Stephens, *Nucleic Acids Res.*, 1990, **18**, 6097–6100.
2 G. E. Crooks, G. Hon, J.-M. Chandonia and S. E. Brenner, *Genome Res.*, 2004, **14**, 1188–1190.
3 J.-D. Chai and M. Head-Gordon, *Phys. Chem. Chem. Phys.*, 2008, **10**, 6615–6620.
4 G. A. Petersson, A. Bennett, T. G. Tensfeldt, M. A. Al-Laham, W. A. Shirley and J. Mantzaris, *J. Chem. Phys.*, 1988, **89**, 2193–2218.
5 G. A. Petersson and M. A. Al-Laham, *J. Chem. Phys.*, 1991, **94**, 6081–6090.
6 G. Lüdemann, P. B. Woiczikowski, T. Kubař, M. Elstner and T. B. Steinbrecher, *J. Phys. Chem. B*, 2013, **117**, 10769–10778.
7 F. Zhang, P. Scheerer, I. Oberpichler, T. Lamparter and N. Krauss, *Proc. Natl. Acad. Sci.*, 2013, **110**, 7217–7222.
8 A. Heck, P. B. Woiczikowski, T. Kubař, K. Welke, T. Niehaus, B. Giese, S. Skourtis, M. Elstner and T. B. Steinbrecher, *J. Phys. Chem. B*, 2014, **118**, 4261–4272.
9 N. Gillet, L. Berstis, X. Wu, F. Gajdos, A. Heck, A. de la Lande, J. Blumberger and M. Elstner, *J. Chem. Theory Comput.*, 2016, **12**, 4793–4805.
10 W. Humphrey, A. Dalke and K. Schulten, *J. Mol. Graph.*, 1996, **14**, 33–38.
11 I. A. Balabin, X. Hu and D. N. Beratan, *J. Comput. Chem.*, 2012, **33**, 906–910.
12 D. N. Beratan, J. N. Betts and J. N. Onuchic, *Science*, 1991, **252**, 1285–1288.
